# 9-Year Trajectory of Depressive and Anxiety Symptoms in Community – the Hong Kong Mental Morbidity Survey Follow Up Studies

**DOI:** 10.1192/bjo.2025.10169

**Published:** 2025-06-20

**Authors:** Linda CW Lam, Bob Z Huo, Owen NW Leung, Allen TC Lee, Eric YH Chen

**Affiliations:** 1 The Chinese University of Hong Kong, Hong Kong SAR, Hong Kong; 2 University of Melbourne, Melbourne, Australia

## Abstract

**Aims:** Depression and anxiety are common in every community. Appreciation of the long-term trajectories of these symptoms will inform more targeted interventions for reduction of disease burden. We evaluated the 7th and 9th year episode onset and remission rates of common mental disorders (CMD) in participants of the Hong Kong Mental Morbidity Survey (HKMMS) at baseline (2010–2023), who were reassessed at 7th and 9th years follow up.

**Methods:** The HKMMS and follow up studies were commissioned by the Medical and Health Research Fund in Hong Kong. Baseline study was conducted from 2010–2013 (n=5,719). We reassessed 1,392 subjects at 7th (2019–2021, COVID pandemic) and 9th (2020–2023, late to post-COVID) years. Depression and anxiety symptoms, episode onset and remission rates of CMD were evaluated with the Clinical Interview Schedule – Revised scores at baseline and follow up. Repeated measures ANCOVA computed factors affecting CISR scores over time.

**Results:** At 7th year, episodes onset rates of CMD in BL normal (n=832, CISR =<5) and BL subsyndromal (n=332, CISR 6–11) were 8.2% and 26.5% respectively. The corresponding figures were 7.9% and 22.3%. Remission rates of CMD from BL (n= 228) to 7th and 9th years were 39.9% and 50.4%. Repeated measure ANCOVA identified a significant time effect with increase in CISR scores at 7th (p<0.001) and decrease from 7th to 9th year (p<0.001). Women had higher CISR at all time points, interaction with time was not significant. Younger age groups (BL 18–44) reported more CISR symptoms at 7th year and greater drop at 9th year. Older adults with physical comorbidity had a trend for increase symptoms over time.

**Conclusion:** In this 9 year follow up study of adults in Hong Kong, episode onsets are related to baseline CISR scores, indicating that psychological symptoms confer long-term risks for deterioration. Conversely, a significant proportion of CMD improved with time. The time frames for assessments unintentionally fell into active COVID pandemic (7th) with social distancing measures, and late to post-COVID pandemic (9th) with lifting of COVID social distancing measures. Our repeated measures evaluation suggested that younger adults are possibly more reactive to environmental stresses. Primary care interventions should consider the age factor into design.

